# Nursing Process for institutionalized older adults: contributions from knowledge awareness workshop

**DOI:** 10.1590/0034-7167-2023-0349

**Published:** 2024-07-29

**Authors:** Francisco Fernandes, Francine Casarin, Patrícia Bitencourt Toscani Greco, Dirce Stein Backes, Oclaris Lopes Munhoz, Silomar Ilha

**Affiliations:** I Universidade Franciscana. Santa Maria, Rio Grande do Sul, Brazil; II Faculdade Integrada de Santa Maria. Santa Maria, Rio Grande do Sul, Brazil; III Universidade Federal do Rio Grande. Rio Grande, Rio Grande do Sul, Brazil; IV Universidade Federal de Santa Maria. Palmeira das Missões, Rio Grande do Sul, Brazil

**Keywords:** Nursing Process, Aged, Homes for the Aged, Knowledge, Nursing, Proceso de Enfermería, Anciano, Hogares para Ancianos, Conocimiento, Enfermería

## Abstract

**Objective:**

To analyze the knowledge of professionals working in a Nursing Home about the Nursing Process before and after the awareness workshop.

**Methods:**

This is strategic action research, developed with nursing professionals and managers of a Nursing Home in Rio Grande do Sul, Brazil. Data were collected between January and June 2023, through semi-structured interviews before and after an awareness workshop. Discursive textual analysis of the data was carried out.

**Results:**

The central category “Understanding about the Nursing Process in Nursing Homes” emerged, which was unitized into two units of meaning and three categories of analysis.

**Conclusion:**

Data revealed non-use and lack of knowledge of the Nursing Process before awareness raising. Afterwards, a deeper understanding of the topic and its importance was identified. Awareness-raising workshops contribute to transformation of knowledge.

## RESUMEN

**Objetivo:**

Analizar el conocimiento de los profesionales que actúan en una Residencia de Larga Estadía sobre el Proceso de Enfermería antes y después del taller de sensibilización.

**Métodos:**

Se trata de una investigación acción estratégica, desarrollada con profesionales de enfermería y gestores de una Residencia de Larga Estadía en Rio Grande do Sul, Brasil. Los datos fueron recolectados entre enero y junio de 2023, mediante entrevistas semiestructuradas antes y después de un taller de sensibilización. Se realizó un análisis textual discursivo de los datos.

**Resultados:**

Surgió la categoría central “Comprensión sobre el Proceso de Enfermería en Residencia de Larga Estadía”, la cual se unificó en dos unidades de significado y tres categorías de análisis.

**Conclusión:**

Los datos revelaron no utilización y desconocimiento del Proceso de Enfermería antes de la sensibilización. Posteriormente se identificó una comprensión más profunda del tema y su importancia. Los talleres de sensibilización contribuyen a la transformación del conocimiento.

## INTRODUCTION

Human aging is a natural process (senescence) in which morphological, physiological and biochemical changes occur in the body, not related to diseases^(1)^. It is characterized as a global phenomenon, since, in 2019, there were 703 million people aged 65 and older in the population, and projections indicate that this number is expected to double to 1.5 billion in 2050. In this regard, there will be two people with over 65 years old for every one up to four years old in the world^(2)^.

Brazil also maintained the aging trend of recent years^(3)^. The country’s total population was estimated, in 2021, at 212.7 million, which represents an increase of 7.6% compared to 2012. During this period, the share of people aged 60 and older increased from 11.3% to 14.7% of the population. In absolute numbers, this age group increased from 22.3 million to 31.2 million, growing 39.8% in the period^(4)^.

Although the aging process does not represent living with diseases, as people age, they become more prone to developing health conditions that require care, which, in the Brazilian reality, are, in most cases, developed or managed by family members. However, depending on the degree of dependence of older adults, family members may present various difficulties in developing or managing care, which may be related to numerous factors, such as the lack of skills to provide care and work routines, which make it difficult for family members to be physically present^(5)^.

Therefore, establishments designed to elder care emerged, among which Nursing Homes (NHs) stand out, which are characterized as public or private places that aim to offer housing, food, assistance with activities of daily living and care by healthcare professionals^(6)^. NHs are establishments providing comprehensive care for people aged 60 and older, dependent or independent, who choose or do not have the means to stay with their family or in a single-cell home^(7)^.

NHs are regulated by Resolution RDC 502 of May 27, 2021 of the Brazilian National Health Regulatory Agency^(8)^. With regard to the technical functioning and modality of assistance services, they can be classified according to different degrees of dependence, namely: dependence I - independent older adults, even if they require the use of adaptive equipment; dependence II - older adults with dependence on up to three self-care activities of daily living, such as food, mobility, hygiene, without cognitive impairment or with controlled cognitive alteration; dependence III - older adults with dependence who require assistance in all self-care activities of daily living and/or with cognitive impairment^(8)^.

In these places, the work of various professionals is evident, with a view to meeting the bio-psycho-socio-spiritual needs of older adults. Among the professionals who work in NHs, the nursing staff stands out, especially nurses, as they are the professionals in charge of leading and managing the care process for people in the different scenarios in which they find themselves^(9-10)^. Furthermore, together with the staff, they spend most of their time caring for older adults.

Nurses’ work with older adults in NH, as well as in other scenarios, must be developed based on the Nursing Process (NP). NP is known as a guiding method for critical thinking and nurses’ clinical judgment, which directs the care developed by the nursing staff to the person, family, community and special groups, through five interrelated, interdependent, recurring and cyclical steps: nursing assessment; nursing diagnosis; nursing planning; nursing implementation; and nursing evolution^(11)^. NP, in care practice, constitutes a method of organizing nursing work, being an important tool in the care process^(12)^.

Considering the necessary use of NP in care, the Federal Nursing Council (COFEN - *Conselho Federal de Enfermagem*), through Resolution 736 of 2024, determined its use throughout the socio-environmental environment in which nursing care occurs^(11)^. However, what is observed in some of these environments is non-execution/application of NP, which denotes the need to understand what may be linked to this situation^(13)^. Therefore, it is necessary to investigate what professionals working in NHs know about NP in elder care, with a view to identifying possible gaps in knowledge, which can be minimized with interventions that aim to deepen knowledge and, therefore, greater possibility of caring for people.

Among the possible methodologies for this purpose, awareness/training workshops stand out, as they provide opportunities for participatory construction, exchange of experiences and knowledge as well as equip participants with the necessary tools. They also enable the construction of groups where participants are willing to work in cooperation, with a view to (re)constructing the situations experienced^(14)^. However, in addition to holding workshops, it is necessary to assess their contribution to professionals, through knowledge production and/or its deepening, a fact that justifies the need and relevance of this research. Furthermore, investigations related to older adults’ health are highlighted by the Ministry of Health (MoH) as research priorities in Brazil^(15)^.

Thus, the question is: what is the knowledge of professionals working in a NH about NP before and after the awareness workshop?

## OBJECTIVE

To analyze the knowledge of professionals working in an NH about NP before and after awareness-raising workshop.

## METHODS

### Ethical aspects

This research is part of a macro project called “Systematization of Nursing Care in an NH: strategic action research”. This project had the general objective of constructing the Systematization of Nursing Care (SAE - *Sistematização da Assistência de Enfermagem*) and NP in an NH. This article addresses one of the specific objectives of such project.

The present study was submitted to the Research Ethics Committee (REC) via *Plataforma Brasil*. Only upon approval by the REC was the first contact made with participants. The ethical and legal precepts involving research with human beings, in accordance with Resolution 466/12 of the MoH, were respected^(16)^. The Informed Consent Form was obtained from all individuals involved in the study in writing. Participant anonymity was maintained, identifying them by the letters “N” (Nurse), “NT” (Nursing Technician) and “M” (Management), followed by a number, according to the interview order (N1, N2...; NT1, NT2...; M1, M2...).

### Study design

This is strategic action research, whose transformation is previously planned by the researcher, who is responsible for monitoring the effects and assessing the results of its application^(17)^. To this end, we followed four phases of action research, divided into eight steps, as can be seen in Chart 1.

**Chart 1:** Schematic representation of action research, Santa Maria, Rio Grande do Sul, Brazil, 2023

ACTION RESEARCH		
**Phase**	**Research steps**	
Diagnosis	Exploratory	Identifying the problem within a context Collecting relevant data
	Diagnosis	Analyzing the data collected Meaning of the data collected Identifying change needs
Planning	Finding possible solutions	
Implementation	Action intervention	
Assessment	Transformation	

*Source: adapted^(18)^.*

To guide the clarity and writing of this report, the checklist COnsolidated criteria for REporting Qualitative research (COREQ)^(19)^ was used.

### Methodological procedures

#### Study setting

This research was developed in a private NH, located in the state of Rio Grande do Sul, Brazil. This institution houses an average of 40 older adults with the purpose of providing adequate care, and is maintained with resources from institutionalized older adults’ families. It has 21 professionals, of which 12 are nursing professionals, four of whom are nurses and eight nursing technicians. Moreover, it has two management professionals, a chief director (also a nurse) and an administrator as well as two physiotherapists, an occupational therapist, a psychologist, a nutritionist and a medical assistant.

#### Data source

The research was carried out with nursing and management professionals from the aforementioned NH. The choice of the nursing staff to participate in this research was made up of professionals in charge of NP execution, organization and supervision, in accordance with Resolution 358/2009, valid during the study period, which was updated and revoked in 2024 by Resolution 736/2024^(11)^. Management professionals were also considered, due to the understanding that, for the success of a work methodology, management support is necessary.

Nurses, nursing technicians and/or management professionals, working in the aforementioned institution for at least three months, a period sufficient for them to have already experienced the reality of the institution were included. Professionals who had a medical certificate or license were excluded. To this end, a convenience sample of ten professionals was constituted.

#### Data collection and organization

Data were collected from the ten participants at two moments. Initially, in January 2023, they were individually invited to participate in the research. Upon acceptance, a semi-structured interview (moment 1) was carried out between January and February 2023, built specifically for this research, which was carried out in a single moment with each participant. The interview guide was composed of two parts; the first, with a description of participants, and the second, with the following open-ended questions: what do you understand by NP? Have you already experienced/developed NP with older adults in the place where you work?

Data collection was conducted by one of the researchers, a male nurse, with clinical and research experience in gerontogeriatrics. The interviews (moment 1) allowed us to verify gaps in knowledge, leading to the identification of needs for change, which led to the need for intervention. Thus, the researchers sought to find possible solutions and, as an intervention, planned a workshop to raise awareness among professionals on issues related to NP, which took place in May 2023. The workshop was held in a conversation round, in the NH itself, lasting an average of one hour. The activity was coordinated by a female nurse, researcher in the area of aging and with experience in SAE in the NH context. It should be noted that the nurse did not have any type of relationship with the participants and did not participate in the data collection stages.

The workshop began with welcoming participants, reaffirming their gratitude for participating in the research. To assist in the activity, audiovisual resources were used, at which time aspects related to NP were addressed, expanding on some points and deepening others, as the need was perceived. Professionals were instructed that, when they felt necessary, they could contribute to the workshop, sharing knowledge and possible experiences. After the nurse’s speech, a conversation about the topic took place, a moment that made it possible to expand and deepen knowledge about the topic at hand.

After the awareness workshop, a new round of semi-structured interviews (moment 2) was held between May and June 2023 with the ten participants to assess knowledge about NP after the intervention using the same instrument used in the first moment. It should be noted that all interviews were, with participants’ authorization, recorded with an MP3 device and then transcribed in full, mechanically, by the researchers, with the help of Microsoft Word^®^ (version 16.31). They were then returned to participants so that they could validate the information.

### Data analysis

**Figure 1:**
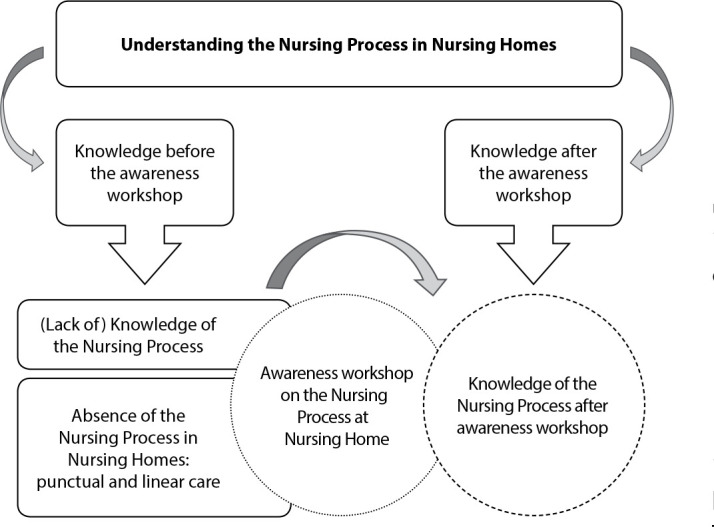
Schematic representation of the central category, units of meaning and categories of analysis

The data from the interviews were subjected to discursive textual analysis, organized based on a recursive sequence of three components: unitarization; establishing relationships; and communication^(20)^. Initially, the researchers examined the texts with intensity and depth, forming the central category by identifying participants’ understanding of NP in NH. Afterwards, each report included in this category was read in detail, being separated into two units of meaning, which gave rise to three categories (Figure 1). Finally, the last stage of the analysis method was carried out, in which the researcher presented the understandings achieved from the two previous focuses, through the communication process, resulting in the metatexts of description and interpretation of the investigated phenomena.

## RESULTS

Of the ten research participants, seven were women and three were men, aged between 37 and 70 years. Regarding training, six were nursing technicians; two were nurses; and two were NH managers, one of them with training in nursing, and the other in administration. As for nursing professionals’ length of training, it ranged from one to 26 years, whereas that of management professionals ranged from 17 to 31 years. Length of professional experience ranged from four months to 20 years in the nursing staff and from 16 to 50 years in the management staff. Nursing professionals’ job tenure ranged from four months to three years, and that of management, from six to nine years.

The analyzed data resulted in a central category, “Understanding about the Nursing Process in Nursing Homes”, which was divided into two units of meaning and three categories of analysis, as shown in Figure 1.

### (Lack of) Knowledge about the Nursing Process

With regard to knowledge about NP before the awareness workshop, it can be seen in statements that nursing technicians were unaware of the topic; even so, they tried to answer based on the analysis of the meaning of the words “process” and “nursing”. Professional nurses and managers presented a slightly more in-depth conception of the subject, although it is still in its infancy.

*I have honestly never heard of the Nursing Process, I believe it is something to contribute, with the pros and cons. You mean medications, care, that kind of thing? Nursing Process is how to act, attitudes, care. It’s knowing what you’re doing, the responsibility you have, it’s your day, literally* [...] *it’s giving the best to your grandparents, general care, medication, hygiene, affection and attention to them.* (NT1)

*I’ve never heard of it* [...] *I think it’s an organization, the standards, is that it? Would it be the way of working, the way of caring* [...] *the way of hygiene, cleaning, things like that? I think that’s it and, like I said, I’ve never heard of it, but I think that’s it. Process of how to work, I wouldn’t know what to say* [...] *I think the Nursing Process is hierarchy, each person’s place, how to behave, I think that’s it.* (NT2)

*I’ll be very honest, I’ve never heard of it, if I have, I haven’t paid attention* [...] *would it be the information technology part, computing, everything that would be typed, like that? Was it treatment, the work part? In this case, I experience my part as a technician, I think it would be the care part, the care we have to take with them, the responsibility we have to have with them, because we are dealing with a life, they are humans.* (NT3)

*The Nursing Process is everything we do* [...] *it is how we put into practice the routine, the obligations, things we do on a daily basis.* (N1)

[...] *I won’t be able to explain this to you, I think it’s a standard, that everyone has to carry out an assessment within a nursing standard. I think it’s similar to the process* [...] *your purpose is to evaluate, to pay full attention, so, within geriatrics, a finer, more delicate look to refine and go a little further, a process that you see when a patient is not cool, then you take a closer look and take action to resolve it. Is that it?* (M1)

*Nursing Process, in my understanding, is the way you will conduct the activities, the name itself says it: process.* (M2)

It can be seen from statements that M1 and M2 had a closer understanding of what is expected with NP, since they described it as being what is carried out, activities that professionals carry out on a daily basis as well as the way of conduct the activities.

### Absence of the Nursing Process in Nursing Homes: punctual and linear care

In the reports of professionals participating in the research, it is possible to notice the presence of a routine elder care in NHs, however the absence of systematized NP in the institution is clear. The responses, in general, were presented in a linear and punctual manner, linked to the procedures and techniques developed on a daily basis with older adults.

*When we bathe, check wounds, apply bandages, change position, help with feeding, administer medication, the care as a whole that the patient needs when they are with us* [...] *assisting each grandmother, doing the routines that are orders, such as putting them to bed, changing diapers, bathing them, giving them supper, before medication, seeing if there is any nebulization to do, checking their temperature* [...] *I think that’s it, doing everything well organized for the next duty, this is all in the process.* (NT2)

*It’s working with what you have within a process, because sometimes you have everything, and sometimes you don’t, so what counts is your view of what’s in front of you* [...] *so, I have to have technique, sensitivity, handling and a view. When I enter the room, I look at everything, I look at the bed, and I think about how I’m going to get the patient out of bed, I see if the patient is well or not, I look at their hair, clothes and feet, and the whole thing, the spiritual part too.* (NT4)

*Care, routine baths, oral hygiene, trichotomies, medications, and signs. And when the patient is very prostrate, they have to pass on to the superior. The nurse decides whether they will be transferred or whether they will stay at the clinic.* (NT5)

*Yes, it would be changing the diaper, managing the diet, keeping it lateralized, that’s what I do* [...] *you have to do everything right away. If I have to administer a diet at eight o’clock, I’ll go there and administer it, I’ll do position changes at the correct time, always placing cushions on the bony prominences to avoid bedsores.* (NT6)

*There’s no way we can’t create a routine* [...] *when I get the patient, I see what they tell me* [...] *for example, I’m going to find a good bed for a grandmother who I know has a pressure ulcer, I’m going to debride it, pass it on to my colleague, because he also has to do it, because, if there isn’t this type of care, this type of planning, systematized, things don’t work out* [...] *as a nurse, you have to have tact, this is care, especially having that view of what is best for patients.* (N2)

### Knowledge about the Nursing Process: perceptions after awareness workshops

After awareness workshops, it was possible to notice a change (transformation) in the knowledge of professionals working in NH about NP, as they expanded and deepened their responses both about the concept and its use.

*In the Nursing Process, the nurse directs us on how to deal with patients daily* [...] *assessing daily, prescribing care, seeing the evolution and what is needed, changing care prescription, if necessary, if it increases or decreases patients’ needs.* (NT1)

*The Nursing Process is the care that technicians develop with that patient based on nurses’ prescriptions and observing daily to see if there is anything more or less to do* [...] *it involves management, unit management and care management.* (NT2)

*It is developed by a nurse, from the moment patients arrive, upon receiving them, assessing them, seeing their history and previous pathologies, it is all part of care management. The Nursing Process includes patient care, such as lying down, changing diapers, and feeding that we, technicians and staff, will develop, following treatment and care prescribed by a nurse.* (NT3)

*These are parameters for us to base on the unit’s nursing care. Based on this, there are SOPs, which help in this process* [...], *it is the nurse who does, organizes what has to be done* [...] *the Nursing Process involves physical examination, patient assessment, interview, see comorbidities and model care, defining conduct, delegating to technicians what to do and how to do it, i.e., they will develop in practice what nurses plan to be carried out. Thus, the Nursing Process has the staff’s help.* (N1)

[...] *the Nursing Process is developed by nurses, with all the steps it has, such as anamnesis and physical examination. Thus, a nurse designates the Nursing Process for nursing technicians to execute, such as care, and nurses supervise and determine the entire process.* (M1)

[...] *the Nursing Process is the issue of care, it involves everyone, the nursing technicians who perform care based on nurses’ prescription.* (M2)

## DISCUSSION

In elder care, it is necessary to train committed professionals who are capable of planning health promotion, prevention and rehabilitation interventions. In this regard, awareness workshops have the potential to contribute to professionals’ knowledge, which increases after participation in training programs. Thus, awareness-raising workshops with healthcare professionals help improve the quality of the care process^(21)^.

In the present research, it was evident that, before the awareness workshop, some of the participants, especially nursing technicians, had never had contact with NP. Nurses and management professionals responded superficially when trying to conceptualize what it meant, referring to NP as nursing obligations and duties or as a standardization in nursing performance. Thus, it is possible to observe the limited knowledge of these professionals regarding a scientific method of nursing work. Similar data was evidenced in an integrative review that describes the fragility that nursing has in assimilating the issues associated with NP^(22)^.

In this review, it was noticed that nurses recognized NP as a methodology that organized nursing practice, i.e., in part, they knew how to conceptualize it. However, they presented a deficit in theoretical, practical and clinical knowledge on the topic^(22)^. Another investigation^(23)^ demonstrated that nurses and nursing technicians recognized the importance of the topic, with evidence of benefits in direct assistance to people, reduction of length of hospitalization, in addition to autonomy in professional practice; however, a lack of knowledge about the concept of NP was observed through the fragmentation of its stages^(23)^.

NP is closely linked to professional identity, as it qualifies and brings benefits to practice. However, there are still some questions regarding its application with regard to conceptual, operational, organizational and political aspects^(24)^.

Regarding the performance of each nursing professional during NP implementation, nurses are exclusively responsible for the nursing diagnosis and prescription stages. Nursing technicians are responsible for taking nursing notes and implementing prescribed care and checking them, under the supervision and guidance of a nurse^(11)^. From this perspective, research carried out in Nigeria states that NP documentation is a global challenge, suggesting the construction of tools to assist in this process^(25)^.

In the present research, it was identified that nursing technicians were unaware of the NP, even if they had tried to respond based on the analysis of the meaning of the words “process” and “nursing”. Professional nurses and managers presented a slightly more in-depth conception of the subject. This data supports what was found in research carried out in southern Minas Gerais, demonstrating the difficulty professionals have in knowing how to use NP, which is negative for quality of care, since lack of knowledge makes planning and implementation difficult of care actions^(26)^.

NP is organized into five stages, such as nursing assessment, nursing diagnosis, nursing planning, nursing implementation and nursing evolution, and must be developed throughout the socio-environmental context in which nursing care occurs^(11)^. A routine of elder care was noticed in this NH, however the presence of NP, effectively, in the institution was not clear. The responses about care in NH, the research setting, referred to the procedures and techniques developed on a daily basis with older adults. These data demonstrate the non-adequate use of NP and imply work without individualized care planning, reflecting elder care in the biological or medical-centered model, not contemplating comprehensiveness.

NP implementation in NH is a necessity in nursing care practice, and it is important to highlight its relevance in elder care, aiming at care quality and patient safety as well as individual care^(27)^. Thus, the importance of implementing a nursing care plan in NH is verified, which must be carried out through the NP^(28)^. Therefore, it is essential to train students and nursing professionals to provide greater sensitivity and support regarding elder care^(29)^.

Hence, awareness workshops stand out as a space for learning, meetings, knowledge production, exchange and expansion of relationships. Therefore, the knowledge constructed in workshops is situated in an experience that can favor important perceptions and raise awareness for human relationships and, more specifically, for the care relationship^(14)^.

In the present research, it was possible to identify the contribution of awareness-raising workshops among NH professionals, since there was a change in professionals’ knowledge about NP, perceived by the expansion and deepening of responses both about the concept and its use. These results converge with a study that carried out awareness-raising workshops to build knowledge in the context of elder care. This study demonstrated that the workshops enabled the expansion and deepening of knowledge on the topic of Alzheimer’s disease (AD) in older adults, perceived by more detailed and coherent responses with what was found in national and international literature on AD after workshops, which can lead to better care for older adults/families^(29)^.

Research carried out on the effects of workshops in the Awareness-Raising Laboratory for training healthcare professionals demonstrated that these contributed to expanding the training of professionals who will deal with care processes in the health-disease aspect, as they enabled the creation of other perceptions and raise-awareness strategies^(14)^. Quasi-experimental study, developed in Cambodia, in Asia, demonstrated the contribution of an intervention to quality nursing care, patient safety and understanding of NP^(30)^.

Therefore, the need to encourage professionals to use interventions, such as awareness-raising workshops in everyday life, stands out, as these can be developed in accordance with management organization and implemented as a permanent education action with employees, or even as specific actions, according to local needs.

### Study limitations

We followed recommendations for conducting and subsequently reporting qualitative research. Even so, assessing the results, based on the interview in a single period after the awareness workshop, may be a limitation of the findings.

### Contributions to nursing, health or public policy

This study demonstrates a contribution to nursing and management in the gerontogeriatric context, as it helped in the process of building knowledge about NP, a fact that could have an impact on elder care in NH. Furthermore, its contribution to science is understood, as it demonstrates the positive result after an intervention made possible through an awareness-raising workshop, which could serve as a model for future research in other realities. Thus, it is suggested that more studies be developed with the aim of assisting in knowledge production about elder care in different contexts.

## FINAL CONSIDERATIONS

This investigation made it possible to analyze nursing professionals’ knowledge and the management of an NH about NP before and after awareness-raising workshops. Before the workshop, lack of knowledge or superficiality regarding the topic was identified. Afterwards, it was possible to identify the expansion of knowledge in both its conceptual and practical aspects. In the conceptual aspect, they were able to describe the meaning of the NP abbreviation. With regard to the concept, the understanding of the NP stood out, as the care itself, based on an assessment that nurses carry out of the needs of older adults in the NH, prescription and guidance of care to be carried out by nursing technicians at older adults. Given the above, the contribution of awareness-raising workshops to transforming the knowledge of professionals working in NH is highlighted, which can contribute to elder care. It is suggested that future research build instruments that assist in NP implementation in NHs as well as assess these tools for a certain period.
