# Supplementary material for: Nursing Process for institutionalized older adults: contributions from knowledge awareness workshop
Source: Rev Bras Enferm. 2024 Jul 29;77(3):e20230349. doi: 10.1590/0034-7167-2023-0349 (PMC11290737; doi:10.1590/0034-7167-2023-0349)
Supplement: Translation

# Translation: Processo de Enfermagem às pessoas idosas institucionalizadas: contribuições de oficina de sensibilização ao conhecimento

**DOI:** 10.1590/0034-7167-2023-0349pt

**Authors:** Francisco Fernandes, Francine Casarin, Patrícia Bitencourt Toscani Greco, Dirce Stein Backes, Oclaris Lopes Munhoz, Silomar Ilha

**Affiliations:** I Universidade Franciscana. Santa Maria, Rio Grande do Sul, Brasil; II Faculdade Integrada de Santa Maria. Santa Maria, Rio Grande do Sul, Brasil; III Universidade Federal do Rio Grande. Rio Grande, Rio Grande do Sul, Brasil; IV Universidade Federal de Santa Maria. Palmeira das Missões, Rio Grande do Sul, Brasil

**Keywords:** Processo de Enfermagem, Idoso, Instituição de Longa Permanência para Idosos, Conhecimento, Enfermagem

## Abstract

**Objetivo:**

Analisar o conhecimento de profissionais atuantes em uma Instituição de Longa Permanência para Idosos sobre o Processo de Enfermagem antes e após oficina de sensibilização.

**Métodos:**

Pesquisa-ação estratégica, desenvolvida com profissionais de enfermagem e gestores de uma Instituição de Longa Permanência para Idosos do Rio Grande do Sul, Brasil. Dados foram coletados entre janeiro e junho de 2023, mediante entrevista semiestruturada antes e após uma oficina de sensibilização. Realizou-se análise textual discursiva dos dados.

**Resultados:**

Emergiu a categoria central “Compreensão acerca do Processo de Enfermagem na Instituição de Longa Permanência para Idosos”, que foi unitarizada em duas unidades de sentido e três categorias de análise.

**Conclusão:**

Dados revelaram a não utilização e o desconhecimento do Processo de Enfermagem antes da sensibilização. Após, identificou-se uma compreensão mais aprofundada sobre a temática e sua importância. Oficinas de sensibilização contribuem à transformação do conhecimento.

## INTRODUÇÃO

O envelhecimento humano é um processo natural (senescência) em que ocorrem alterações morfológicas, fisiológicas, bioquímicas no organismo, não relacionadas com doenças^(1)^. Caracteriza-se como um fenômeno mundial, já que, em 2019, havia 703 milhões de pessoas com 65 anos ou mais na população, e projeções indicam que esse número deverá dobrar para 1,5 bilhão em 2050. Desse modo, haverá duas pessoas com mais de 65 anos para cada uma de até quatro anos no mundo^(2)^.

O Brasil também manteve a tendência de envelhecimento dos últimos anos^(3)^. A população total do país foi estimada, no ano de 2021, em 212,7 milhões, o que representa um aumento de 7,6% em relação ao ano de 2012. Nesse período, a parcela de pessoas com 60 anos ou mais se elevou de 11,3% para 14,7% da população. Em números absolutos, esse grupo etário passou de 22,3 milhões para 31,2 milhões, crescendo 39,8% no período^(4)^.

Embora o processo de envelhecimento não represente (con)viver com doenças, à medida que as pessoas envelhecem, tornam-se mais propensas ao desenvolvimento de condições de saúde que exigem cuidados, os quais, na realidade brasileira, são, na maioria nas vezes, desenvolvidos ou gerenciados por familiares. No entanto, conforme o grau de dependência da pessoa idosa, os familiares podem apresentar dificuldades diversas para o desenvolvimento ou gerenciamento do cuidado, as quais podem estar relacionadas a inúmeros fatores, como à ausência de habilidades para realizar o cuidado e às rotinas de trabalho, que dificultam a presença física dos familiares^(5)^.

Logo, surgem estabelecimentos destinados ao cuidado das pessoas idosas, dentre os quais destacam-se as Instituições de Longa Permanência para Idosos (ILPIs), as quais se caracterizam como locais públicos ou privados que possuem o objetivo de oferecer a moradia, alimentação, auxílio em atividades diárias e cuidado por profissionais de saúde^(6)^. As ILPIs são estabelecimentos para atendimento integral de pessoas com 60 anos ou mais, dependentes ou independentes, que optam ou que não dispõem de condições para permanecer com a família ou em domicílio unicelular^(7)^.

As ILPIs são regulamentadas pela Resolução RDC nº 502, de 27 de maio de 2021, da Agência Nacional de Vigilância Sanitária^(8)^. No que se refere ao funcionamento técnico e à modalidade dos serviços assistenciais, podem ser classificadas de acordo com os diferentes graus de dependência, quais sejam: dependência I - pessoas idosas independentes, mesmo que requeiram uso de equipamentos de autoajuda; dependência II - pessoas idosas com dependência em até três atividades de autocuidado para a vida diária, tais como alimentação, mobilidade, higiene, sem comprometimento cognitivo ou com alteração cognitiva controlada; dependência III - pessoas idosas com dependência que requeiram assistência em todas as atividades de autocuidado para a vida diária e/ou com comprometimento cognitivo^(8)^.

Nesses locais, denota-se a atuação de profissionais variados, com vistas a atender às necessidades bio-psico-socio-espirituais das pessoas idosas. Entre os profissionais que atuam na ILPI, destaca-se a equipe de enfermagem, em especial o enfermeiro, por ser o profissional responsável pela liderança e gerenciamento do processo de cuidado às pessoas nos diferentes cenários em que essas se encontram^(9-10)^. Além disso, junto à equipe, permanece a maior parte do tempo nos cuidados às pessoas idosas.

A atuação do enfermeiro junto às pessoas idosas na ILPI, assim como em outros cenários, deve ser desenvolvida com base no Processo de Enfermagem (PE). PE é conhecido como método orientador do pensamento crítico e o julgamento clínico do enfermeiro, o qual direciona o cuidado desenvolvido pela equipe de enfermagem à pessoa, família, coletividade e grupos especiais, por meio de cinco etapas inter-relacionadas, interdependentes, recorrentes e cíclicas: avaliação de enfermagem; diagnóstico de enfermagem; planejamento de enfermagem; implementação de enfermagem; e evolução de enfermagem^(11)^. O PE, na prática assistencial, constitui-se um método de organização do trabalho de enfermagem, sendo uma importante ferramenta no processo do cuidado^(12)^.

Tendo em vista a necessária utilização do PE no cuidado, o Conselho Federal de Enfermagem (COFEN), por meio da Resolução n^o^. 736 de 2024, determinou o uso do mesmo em todo o ambiente socioambiental em que ocorra o cuidado de enfermagem^(11)^. Entretanto, o que se observam em alguns desses ambientes são a não execução/aplicação do PE, o que denota a necessidade de compreender o que pode estar atrelado a essa situação^(13)^. Assim, é preciso investigar o que os profissionais que atuam em ILPIs conhecem sobre o PE no cuidado à pessoa idosa, com vistas à identificação de possíveis lacunas do conhecimento, as quais podem ser minimizadas com intervenções que objetivem aprofundar o conhecimento e, por conseguinte, maior possibilidade de cuidado às pessoas.

Entre as metodologias possíveis para esse fim, destacam-se as oficinas de sensibilização/capacitação, pois oportunizam a construção participativa, a troca de experiências e conhecimentos, assim como instrumentalizam os participantes. Possibilitam, ainda, a construção de grupos onde os participantes se dispõem a trabalhar em cooperação, com vistas à (re)construção das situações vivenciadas^(14)^. Contudo, para além de realizar oficinas, é preciso avaliar a contribuição destas para os profissionais, por meio da produção de conhecimento e/ou aprofundamento do mesmo, fato que justifica a necessidade e relevância desta pesquisa. Além disso, investigações relacionadas à saúde da pessoa idosa são destacadas pelo Ministério da Saúde (MS) como prioridades de pesquisa no Brasil^(15)^.

Nesse sentido, questiona-se: qual o conhecimento de profissionais atuantes em uma instituição de longa permanência para idosos sobre o Processo de Enfermagem antes e após oficina de sensibilização?

## OBJETIVO

Analisar o conhecimento de profissionais atuantes em uma instituição de longa permanência para idosos sobre o Processo de Enfermagem antes e após oficina de sensibilização.

## MÉTODOS

### Aspectos éticos

Esta pesquisa faz parte de um macroprojeto denominado “Sistematização da Assistência de Enfermagem em uma instituição de longa permanência para idosos: pesquisa-ação estratégica”. O referido projeto teve o objetivo geral de construir a SAE e o PE em uma ILPI. Este artigo contempla um dos objetivos específicos do projeto.

O presente estudo foi submetido à apreciação de um Comitê de Ética em Pesquisa com Seres Humanos (CEP) via Plataforma Brasil. Somente após a aprovação do CEP, realizou-se o primeiro contato com os participantes. Consideraram-se os preceitos éticos e legais que envolvem a pesquisa com seres humanos, conforme a Resolução n^o^. 466/12 do MS^(16)^. O Termo de Consentimento Livre e Esclarecido foi obtido de todos os indivíduos envolvidos no estudo por meio escrito. Manteve-se o anonimato dos participantes, identificando-os pelas letras “E” (Enfermeiro), “TE” (Técnico de Enfermagem) e “G” (Gestão), seguidas por um algarismo, conforme a ordem de entrevista (E1, E2...; TE1, TE2...; G1, G2...).

### Tipo de estudo

Trata-se de pesquisa-ação estratégica, cuja transformação é previamente planejada pelo pesquisador, o qual é o responsável por acompanhar os efeitos e avaliar os resultados de sua aplicação^(17)^. Para tanto, seguiram-se quatro fases da pesquisa-ação, dividida em oito passos, conforme pode ser visualizado no Quadro 1.

**Quadro 1:** Representação esquemática da pesquisa-ação, Santa Maria, Rio Grande do Sul, Brasil, 2023

PESQUISA-AÇÃO		
**Fase**	**Passos da pesquisa**	
Diagnóstica	Exploratória	Identificação do problema dentro de um contexto Levantamento dos dados pertinentes
	Diagnóstica	Análise dos dados levantados Significado dos dados levantados Identificação das necessidades de mudança
Planejamento	Encontrando possíveis soluções	
Implementação	Intervenção-ação	
Avaliação	Transformação	

*Fonte: adaptado^(18)^.*

Para nortear a clareza e redação deste relatório, utilizou-se o *checklist COnsolidated criteria for REporting Qualitative research* (COREQ)^(19)^.

### Procedimento metodológicos

#### Cenário do estudo

Pesquisa desenvolvida em uma ILPI privada, localizada no estado do Rio Grande do Sul, Brasil. A referida instituição abriga em média 40 pessoas idosas com a finalidade de proporcionar um cuidado adequado, e se mantém com recursos dos familiares dos institucionalizados. Possui 21 profissionais, dos quais 12 são profissionais de enfermagem, sendo quatro enfermeiros e oito técnicas(os) de enfermagem. Além disso, conta com dois profissionais da gestão, sendo um diretor-chefe (também enfermeiro) e uma administradora, além de duas fisioterapeutas, uma terapeuta ocupacional, uma psicóloga, uma nutricionista e um médico assistencial.

#### Fonte de dados

A pesquisa foi realizada com profissionais de enfermagem e gestão da referida ILPI. A escolha da equipe de enfermagem para participar desta pesquisa adveio de ser composta por profissionais responsáveis pela execução, organização e supervisão do PE, conforme a Resolução n^o^. 358/2009, válida no período do estudo, a qual foi atualizada e revogada no ano de 2024 pela Resolução n^o^. 736/2024^(11)^. Os profissionais da gestão foram também considerados, pela compreensão de que, para o sucesso de uma metodologia de trabalho, é necessário o apoio da gestão.

Incluíram-se enfermeiros, técnicos de enfermagem e/ou profissionais da gestão, atuantes na referida instituição por no mínimo três meses, período suficiente para que já tenham vivenciado e experienciado a realidade da instituição. Excluíram-se os profissionais que estavam de atestado médico ou licença. Para tanto, constituiu-se uma amostragem por conveniência de 10 profissionais.

#### Coleta e organização dos dados

Os dados foram coletados com os 10 participantes em dois momentos. Inicialmente, no mês de janeiro de 2023, eles foram convidados individualmente a participar da pesquisa. Após o aceite, foi realizada, entre janeiro e fevereiro de 2023, a entrevista (momento 1) semiestruturada, construída especificamente para esta pesquisa, sendo esta desenvolvida em um único momento com cada participante. O roteiro de entrevista foi composto de duas partes; a primeira, com a descrição dos participantes, e a segunda, com as seguintes questões abertas: o que você compreende por PE? Você já vivenciou/desenvolveu o PE junto a pessoas idosas no local em que você atua?

A coleta dos dados foi conduzida por um dos pesquisadores, enfermeiro, do sexo masculino, com experiência clínica e de pesquisa na área da gerontogeriatria. As entrevistas (momento 1) permitiram verificar lacunas do conhecimento, conduzindo à identificação das necessidades de mudança, o que despertou para a necessidade da intervenção. Assim, os pesquisadores buscaram encontrar possíveis soluções e, como intervenção, planejaram uma oficina com vistas à sensibilização dos profissionais sobre questões relativas ao PE, que ocorreu no mês de maio de 2023. A oficina foi realizada a partir de uma roda de conversa, na própria ILPI, com duração média de uma hora. A atividade foi coordenada por uma enfermeira, do sexo feminino, pesquisadora na área do envelhecimento e com experiência em SAE no contexto da ILPI. Salienta-se que a enfermeira não possuía nenhum tipo de relação com os participantes e não participou das etapas de coleta dos dados.

Iniciou-se a oficina com as boas-vindas aos participantes, reafirmando o agradecimento pela participação na pesquisa. Para auxiliar na atividade, utilizou-se recurso audiovisual, momento em que foram abordados os aspectos relacionados ao PE, ampliando alguns pontos e aprofundando outros, conforme percebia-se a necessidade. Os profissionais foram orientados de que, quando sentissem necessidade, poderiam contribuir com a oficina, compartilhando conhecimentos e possíveis experiências. Após a fala da enfermeira, ocorreu a roda de conversa sobre a temática, momento em que possibilitou ampliar e aprofundar o conhecimento sobre a temática em tela.

Após a oficina de sensibilização, realizou-se, entre maio e junho de 2023, nova rodada de entrevistas semiestruturadas (momento 2) com os 10 participantes, com vistas à avaliação do conhecimento sobre o PE após a intervenção utilizando-se o mesmo instrumento empregado no primeiro momento. Salienta-se que todas as entrevistas foram, com a autorização dos participantes, gravadas com aparelho MP3 e, após, transcritas na íntegra, mecanograficamente, pelos pesquisadores, com o auxílio do programa *Microsoft Word^®^* (versão 16.31). Na sequência, foram devolvidas aos participantes, para que eles validassem as informações.

#### Análise dos dados

Os dados provenientes das entrevistas foram submetidos à análise textual discursiva, organizada a partir de uma sequência recursiva de três componentes: unitarização; estabelecimento de relações; e comunicação^(20)^. Inicialmente, os pesquisadores examinaram os textos com intensidade e profundidade, formando a categoria central a partir da identificação do entendimento dos participantes acerca do PE na ILPI. Após, cada relato inserido nesta categoria foi lido de forma minuciosa, sendo separado em duas unidades de sentido, das quais originaram três categorias (Figura 1). Por fim, procedeu-se à última etapa do método de análise, na qual o pesquisador apresentou as compreensões atingidas a partir dos dois focos anteriores, pelo processo de comunicação, resultando nos metatextos de descrição e interpretação dos fenômenos investigados.

**Figura 1:** Representação esquemática da categoria central, unidades de sentido e das categorias de análise

## RESULTADOS

Dos 10 participantes da pesquisa, sete eram mulheres e três eram homens, com idades entre 37 e 70 anos. Quanto à formação, seis eram técnicos de enfermagem; dois eram enfermeiros; e dois eram gestores da ILPI, um deles com formação em enfermagem, e o outro, em administração. No que se refere ao tempo de formação dos profissionais da enfermagem, variou de um a 26 anos, enquanto o dos profissionais da gestão variou de 17 a 31 anos. O tempo de experiência profissional variou de quatro meses a 20 anos na equipe de enfermagem e de 16 a 50 anos da equipe da gestão. O tempo de atuação dos profissionais de enfermagem no serviço variou de quatro meses a três anos, e o da gestão, de seis a nove anos.

Os dados analisados resultaram em uma categoria central, “Compreensão acerca do Processo de Enfermagem na Instituição de Longa Permanência para Idosos”, a qual foi unitarizada em duas unidades de sentido e três categorias de análise, conforme a Figura 1.

### (Des)Conhecimento sobre o Processo de Enfermagem

No que se refere ao conhecimento acerca do PE antes da oficina de sensibilização, pode-se perceber nas falas que os técnicos de enfermagem apresentavam desconhecimento acerca da temática; mesmo assim, tentaram responder com base na análise do significado das palavras “processo” e “enfermagem”. Os profissionais enfermeiros e gestores apresentaram uma concepção um pouco mais aprofundada acerca do assunto, contudo ainda incipiente.

*Processo de Enfermagem, sinceramente, nunca ouvi falar, acredito que seja algo para contribuir, com os prós e contras. Você quer dizer que medicamentos, cuidados, esse tipo de coisa? Processo de Enfermagem é como agir, as atitudes, é os cuidados. É saber o que está fazendo, responsabilidade que você tem, é o teu dia, literalmente* [...] *é dar o melhor para os avôs, os cuidados em geral, medicação, higiene, carinho e atenção com eles.* (TE1)

*Nunca ouvi falar* [...] *penso que seja uma organização, os padrões, é isso? Seria o jeito de trabalhar, o jeito de cuidar* [...] *o jeito com a higienização, com a limpeza, essas coisas assim? Eu acho que é isso e, como eu disse, eu nunca ouvi falar, mas acho que é isso. Processo de como trabalhar, não saberia o que dizer* [...] *eu acho que o processo da enfermagem é hierarquia, o lugar de cada um, como se portar, acho que é isso.* (TE2)

*Vou ser bem sincera, nunca ouvi falar, se eu ouvi, não prestei atenção* [...] *seria a parte da informática, da computação, tudo o que seria digitado, tipo assim? Seria o tratamento, a parte do trabalho? No caso, eu vivencio a minha parte de técnico, eu acho que seria a parte dos cuidados, o cuidado que temos que ter com eles, a responsabilidade que a gente tem que ter com eles, porque a gente está lidando com uma vida, são humanos.* (TE3)

*Processo de Enfermagem é tudo o que a gente realiza* [...] *é como a gente vai pôr em prática a rotina, as obrigações, coisas que faz no dia a dia.* (E1)

[...] *não vou conseguir te explicar isso, eu penso que é um padrão, que todo mundo tem que fazer uma avaliação dentro de um padrão da enfermagem. Eu acho que é semelhante ao processo* [...] *o propósito de vocês é avaliar, ter toda atenção, assim, dentro da geriatria, um olhar mais fino, mais delicado para apurarem e irem um pouco mais longe, um processo que tu vês quando o cliente não está legal, daí tu correr aquele olho e já tomar as atitudes para resolver. É isso?* (G1)

*Processo de Enfermagem, no meu entendimento, é a forma como tu vai conduzir as atividades, o próprio nome já diz: processo.* (G2)

Pode-se perceber pelas falas que E1 e G2 possuíam uma compreensão mais próxima do que se espera com o PE, uma vez que o descreveram como sendo aquilo que se realiza, atividades que os profissionais desenvolvem no dia a dia, bem como a forma de conduzir as atividades.

### Ausência do Processo de Enfermagem na Instituição de Longa Permanência para Idosos: cuidado pontual e linear

Nos relatos dos profissionais participantes da pesquisa, pode-se perceber a presença de uma rotina de assistência à pessoa idosa na ILPI, contudo fica clara a ausência do PE sistematizado na instituição. As respostas, de modo geral, apresentaram-se de forma linear e pontual, atreladas aos procedimentos e técnicas desenvolvidos no cotidiano junto às pessoas idosas.

*Quando a gente dá banho, verifica as feridas, faz curativo, mudança de decúbito, auxilia na alimentação, administra medicação, os cuidados como um todo que o paciente necessita quando está conosco* [...] *atender cada vó, fazendo as rotinas que são mandadas, como colocar na cama, trocar fraldas, dar banho, dar ceia, antes da medicação, ver se tem alguma nebulização para fazer, verificar se tem temperatura* [...] *eu acho que é isso, fazer tudo bem organizado para o próximo plantão, está tudo isto no processo.* (TE2)

*É trabalhar com o que se tem dentro de um processo, porque às vezes tem tudo, e às vezes, não, daí o que vale é a tua visão perante o que está na sua frente* [...] *então, eu tenho que ter a técnica, a sensibilidade, o manejo e uma visão. Quando eu entro no quarto, eu já vou olhando tudo, eu olho a cama, e penso como vou tirar o paciente da cama, vejo se o paciente está bem ou não, olho seu cabelo, a roupa e os pés, e o conjunto todo, a parte espiritual também.* (TE4)

*Os cuidados, os banhos de rotina, as higienizações via oral, as tricotomias, as medicações, e os sinais. E quando o paciente está muito prostrado, tem que passar adiante para o superior. O enfermeiro resolver se será transferido ou se vai ficar na clínica.* (TE5)

*Sim, seria a troca de fralda, a administração da dieta, deixar lateralizado, é isso o que eu faço* [...] *tem que fazer tudo na hora. Se tem que administrar dieta às oito horas, vou lá e administro, vou fazer as trocas de decúbito no horário correto, sempre colocando almofadas nas proeminências ósseas para evitar escara.* (TE6)

*Não tem como a gente não criar uma rotina* [...] *quando eu pego o paciente, eu vejo o que ele me refere* [...] *por exemplo, vou ver uma boa cama para uma vó que eu sei que tem uma úlcera por pressão, vou desbridar, passar para o meu colega, que ele também tem que fazer, porque, se não tiver este tipo de cuidado, esse tipo de planejamento, sistematizado, a coisa não anda.* [...] *como enfermeiro, tem que ter o tato, esse é o cuidado, principalmente ter aquela visão do que é melhor para o paciente.* (E2)

### Conhecimento acerca do Processo de Enfermagem: percepções após as oficinas de sensibilização

Após as oficinas de sensibilização, pôde-se perceber uma mudança (transformação) no conhecimento dos profissionais atuantes na ILPI sobre o PE, pois ampliaram e aprofundaram suas respostas tanto sobre o conceito quanto sobre a sua utilização.

*No Processo de Enfermagem, o enfermeiro nos direciona para como lidar com o paciente diariamente* [...] *avaliando diariamente, prescrevendo cuidados, vendo a evolução e o que é necessário, mudando a prescrição de cuidados, se for necessário, caso aumente ou diminua a necessidade do paciente.* (TE1)

*O Processo de Enfermagem é o cuidado que o técnico desenvolve com aquele paciente a partir das prescrições do enfermeiro e observando diariamente para ver se tem alguma coisa para fazer a mais ou a menos* [...] *envolve o gerenciamento, a gestão da unidade e do cuidado.* (TE2)

*É desenvolvido pelo enfermeiro, desde que o paciente chega, ao recebê-lo, avaliá-lo, ver sua história e patologias prévias, é toda a parte da gestão do cuidado. No Processo de Enfermagem, entram os cuidados aos pacientes, como os decúbitos, trocas de fraldas, alimentação que nós, técnicos e equipe, vamos desenvolver, seguindo o tratamento e cuidados prescritos pelo enfermeiro.* (TE3)

*São parâmetros pra gente se basear na assistência de enfermagem da unidade. Baseado nisso, têm-se os POPs, que auxiliam nesse processo* [...], *é o enfermeiro quem faz, organiza o que tem que ser feito* [...] *o Processo de Enfermagem envolve exame físico, avaliação do paciente, a entrevista, ver as comorbidades e modelar os cuidados, definindo as condutas, delegando ao técnico o que fazer e como fazer, ou seja, eles irão desenvolver na prática o que o enfermeiro programar para ser realizado. Assim, o Processo de Enfermagem tem o auxílio da equipe.* (E1)

[...] *o Processo de Enfermagem é desenvolvido pelos enfermeiros, com todos os passos que ele possui, exemplo: anamnese e exame físico. Assim, o enfermeiro designa o Processo de Enfermagem para os técnicos de enfermagem o executarem, como os cuidados, e os enfermeiros supervisionam e determinam todo o processo.* (G1)

[...] *o Processo de Enfermagem é a questão do cuidado, envolve todo mundo, os técnicos de enfermagem que executam os cuidados a partir da prescrição do enfermeiro.* (G2)

## DISCUSSÃO

No cuidado à pessoa idosa, faz-se necessária a formação de profissionais comprometidos que sejam capazes de planejar intervenções de promoção, prevenção e reabilitação à saúde. Nesse sentido, oficinas de sensibilização possuem o potencial de contribuir com o conhecimento dos profissionais, o qual aumenta após a participação em programas de treinamento. Assim, as oficinas de sensibilizações com profissionais da área da saúde auxiliam na qualidade do processo de cuidado^(21)^.

Na presente pesquisa, evidenciou-se que, antes da oficina de sensibilização, parte dos participantes, especialmente os técnicos de enfermagem, nunca havia tido contato com a temática do PE. Já os enfermeiros e os profissionais da gestão responderam de forma superficial ao tentarem conceituar o que o mesmo significava, referindo-se ao PE como obrigações e deveres da enfermagem ou como uma padronização na atuação da enfermagem. Assim, é possível observar a limitação de conhecimento desses profissionais acerca de um método científico de trabalho da enfermagem. Dado semelhante foi evidenciado em revisão integrativa que descreve a fragilidade que a enfermagem possui para assimilar as questões associadas ao PE^(22)^.

Nesta revisão, percebeu-se que os enfermeiros reconheciam o PE como uma metodologia que organizava a prática de enfermagem, ou seja, em parte, sabiam conceituá-lo. Contudo, apresentaram déficit de conhecimento teórico, prático e clínico acerca da temática^(22)^. Outra investigação^(23)^ demonstrou que os enfermeiros e técnicos de enfermagem reconheciam a importância da temática, com evidências de benefícios na assistência direta às pessoas, redução do tempo de permanência em internação, além de autonomia no exercício profissional; porém, foi observado déficit de conhecimento sobre o conceito de PE através da fragmentação das suas etapas^(23)^.

O PE está intimamente ligado à identidade profissional, uma vez que qualifica e traz benefícios para a prática. Entretanto, ainda perpassam alguns questionamentos a respeito da sua aplicação no que diz respeito aos aspectos conceituais, operacionais, organizacionais e políticos^(24)^.

No que se refere à atuação de cada profissional de enfermagem durante a efetivação do PE, cabe privativamente aos enfermeiros as etapas de diagnóstico e prescrição de enfermagem. Ao técnico em enfermagem, compete atuar na realização de anotações de enfermagem e na implementação dos cuidados prescritos e sua checagem, sob a supervisão e orientação do enfermeiro^(11)^. Nessa perspectiva, investigação desenvolvida na Nigéria refere ser um desafio global a documentação do PE, sugerindo a construção de ferramentas para auxiliar nesse processo^(25)^.

Na presente pesquisa, identificou-se que os técnicos de enfermagem desconheciam o PE, mesmo que tivessem tentado responder com base na análise do significado das palavras “processo” e “enfermagem”. Os profissionais enfermeiros e gestores apresentaram uma concepção um pouco mais aprofundada acerca do assunto. Esse dado corrobora o encontrado em pesquisa desenvolvida na região Sul do estado de Minas Gerais, demonstrando a dificuldade de conhecimento dos profissionais na utilização do PE, o que é negativo para a qualidade do cuidado, uma vez que o desconhecimento dificulta o planejamento e a implementação das ações de cuidado^(26)^.

O PE organiza-se em cinco etapas, como avaliação de enfermagem, diagnóstico de enfermagem, planejamento de enfermagem, implementação e evolução de enfermagem, e deve ser desenvolvido em todo o contexto socioambiental em que ocorra o cuidado de enfermagem^(11)^. Percebeu-se nesta ILPI uma rotina de assistência à pessoa idosa, contudo não ficou clara a presença do PE, efetivamente, na instituição. As respostas sobre o cuidado na ILPI, cenário da pesquisa, referiram-se aos procedimentos e técnicas desenvolvidos no cotidiano junto às pessoas idosas. Esses dados demonstram a não utilização adequada do PE e implicam um trabalho sem planejamento individualizado da assistência, refletindo em um cuidado à pessoa idosa no modelo biologicista ou médico-centrado, não contemplando a integralidade.

A execução do PE em ILPI é uma necessidade na prática assistencial do enfermeiro, sendo importante ressaltar sua relevância junto à assistência da pessoa idosa, visando à qualidade e à segurança do paciente, bem como à individualidade do cuidado^(27)^. Assim, verifica-se a importância da implantação de um plano de cuidados de enfermagem em ILPI, o qual deve ser efetivado por meio do PE^(28)^. Dessa forma, torna-se imprescindível a capacitação de estudantes e dos profissionais de enfermagem para maior sensibilidade e embasamento no que tange ao cuidado à pessoa idosa^(29)^.

Nesse sentido, as oficinas de sensibilização destacam-se como um espaço de aprendizagem, encontros, produção de conhecimentos, intercâmbio e ampliação das relações. Logo, o conhecimento construído nas oficinas se situa em uma experiência que pode favorecer percepções e sensibilidades importantes para as relações humanas e, mais especificamente, para a relação de cuidado^(14)^.

Na presente pesquisa, foi possível identificar a contribuição da oficina de sensibilização junto aos profissionais da ILPI, uma vez que ocorreu a mudança de conhecimento dos profissionais sobre o PE, percebida pela ampliação e aprofundamento das respostas tanto sobre o conceito quanto sobre a utilização. Esses resultados convergem com estudo que realizou oficinas de sensibilização para a construção do conhecimento no contexto de cuidados das pessoas idosas. Tal estudo demonstrou que as oficinas possibilitaram a ampliação e o aprofundamento do conhecimento sobre a temática da doença de Alzheimer (DA) na pessoa idosa, percebido por respostas mais detalhadas e coerentes com o encontrado na literatura nacional e internacional sobre a DA após as oficinas, o que pode conduzir a um melhor cuidado às pessoas idosas/famílias^(29)^.

Pesquisa realizada sobre os efeitos das oficinas no Laboratório de Sensibilidades para a formação profissionais de saúde demonstrou que essas contribuíram para ampliar a formação do profissional que lidará com os processos de cuidado do aspecto saúde-doença, pois possibilitaram a criação de outras percepções e sensibilidades^(14)^. Estudo quase-experimental, desenvolvido em Camboja, no continente asiático, demonstrou a contribuição de uma intervenção para a qualidade dos cuidados de enfermagem, segurança do paciente e compreensão do PE^(30)^.

Dessa forma, salienta-se a necessidade de estimular profissionais para a utilização de intervenções, como as oficinas de sensibilização no cotidiano, pois essas poderão ser desenvolvidas de acordo com a organização da gestão e implementadas como ação de educação permanente junto aos colaboradores, ou ainda como ações pontuais, conforme a necessidade local.

### Limitações do estudo

Foram seguidas as recomendações para a condução e posterior relatório de pesquisas qualitativas. Mesmo assim, a realização da avaliação dos resultados, a partir da entrevista em um único período após a oficina de sensibilização, pode ser uma limitação dos achados.

### Contribuições para a área da enfermagem, saúde ou políticas públicas

Este estudo demonstra contribuição para a enfermagem e gestão no contexto gerontogeriátrico, uma vez que auxiliou no processo de construção de conhecimentos sobre o PE, fato que poderá repercutir no cuidado às pessoas idosas na ILPI. Além disso, compreende-se sua contribuição para a ciência, pois demonstra o resultado positivo após uma intervenção possibilitada a partir de uma oficina de sensibilização, a qual poderá servir de modelo para pesquisas futuras em outras realidades. Assim, sugere-se que mais estudos sejam desenvolvidos com o intuito de auxiliar na produção de conhecimento sobre o cuidado à pessoa idosa em diferentes contextos.

## CONSIDERAÇÕES FINAIS

Esta investigação possibilitou analisar o conhecimento dos profissionais de enfermagem e da gestão de uma ILPI acerca do PE antes e após oficinas de sensibilização. Antes da oficina, identificou-se o desconhecimento ou superficialidade acerca da temática. Após, foi possível identificar a ampliação do conhecimento tanto no seu aspecto conceitual quanto prático. No aspecto conceitual, souberam descrever o significado da abreviação do PE. No que se refere ao conceito, destacou-se a compreensão do PE, como o cuidado em si, a partir de uma avaliação que o enfermeiro realiza das necessidades das pessoas idosas na ILPI, prescrição e orientação dos cuidados a serem realizados pelos técnicos de enfermagem à pessoa idosa. Diante do exposto, denota-se a contribuição das oficinas de sensibilização para a transformação do conhecimento dos profissionais atuantes na ILPI, o que pode contribuir com o cuidado direcionado às pessoas idosas. Sugere-se que pesquisas futuras construam instrumentos que auxiliem na implementação do PE em ILPIs, bem como avaliem por determinado período essas ferramentas.
